# A Specific Association between Facial Disgust Recognition and Estradiol Levels in Naturally Cycling Women

**DOI:** 10.1371/journal.pone.0122311

**Published:** 2015-04-15

**Authors:** Sunjeev K. Kamboj, Kathleen M. Krol, H. Valerie Curran

**Affiliations:** Research Department of Clinical, Educational and Health Psychology, University College London, London, United Kingdom; University of Tuebingen Medical School, GERMANY

## Abstract

Subtle changes in social cognition are associated with naturalistic fluctuations in estrogens and progesterone over the course of the menstrual cycle. Using a dynamic emotion recognition task we aimed to provide a comprehensive description of the association between ovarian hormone levels and emotion recognition performance using a variety of performance metrics. Naturally cycling, psychiatrically healthy women attended a single experimental session during a follicular (days 7–13; n = 16), early luteal (days 15–19; n = 14) or late luteal phase (days 22–27; n = 14) of their menstrual cycle. Correct responses and reaction times to dynamic facial expressions were recorded and a two-high threshold analysis was used to assess discrimination and response bias. Salivary progesterone and estradiol were assayed and subjective measures of premenstrual symptoms, anxiety and positive and negative affect assessed. There was no interaction between cycle phase (follicular, early luteal, late luteal) and facial expression (sad, happy, fearful, angry, neutral and disgusted) on any of the recognition performance metrics. However, across the sample as a whole, progesterone levels were positively correlated with reaction times to a variety of facial expressions (anger, happiness, sadness and neutral expressions). In contrast, estradiol levels were *specifically* correlated with disgust processing on three performance indices (correct responses, response bias and discrimination). Premenstrual symptoms, anxiety and positive and negative affect were not associated with emotion recognition indices or hormone levels. The study highlights the role of naturalistic variations in ovarian hormone levels in modulating emotion recognition. In particular, progesterone seems to have a general slowing effect on facial expression processing. Our findings also provide the first behavioural evidence of a specific role for estrogens in the processing of disgust in humans.

## Introduction

The capacity for understanding intentional mental states in other people (i.e. their beliefs, desires and intentions) is a cornerstone of co-operation, intimacy and general adaptive interpersonal functioning. A critical component of this capability involves decoding facial expressions of emotion in others, an ability which is formed early in human development and is exquisitely honed by adolescence, when decoding of subtle interpersonal cues becomes essential to adaptive functioning in communities. Derangement of this capacity for mentalizing is a central feature of psychopathology [1] and may arise from adverse developmental experiences [2]. It is therefore critical to determine the factors influencing this capability and those involved in its dysregulation. One approach to understanding basic aspects of mentalizing has involved parsing the social and biological determinants of performance on emotion-recognition tasks.

Gender is one such determinant. However, while women tend to out-perform men on emotion recognition tasks [3–5], their performance appears to vary as a function of circulating ovarian hormone levels (see below). Several applied and theoretical implications follow from this observation. Firstly, given that ovarian hormones fluctuate across the menstrual cycle [6], changes in affect recognition ability may be detectable across different phases of the cycle. Secondly, some researchers have hypothesised that changes in emotional processing and social decision-making across the menstrual cycle and during pregnancy reflect evolutionarily important adaptations in emotional competence driven by changes in ovarian hormones that enhance the pre- and postnatal survival chances of mother and infant in the face of environmental (especially interpersonal-) threats [7]. Thirdly, changes in emotion recognition ability may reflect the formation and expression of cognitive-emotional biases such as those seen in anxiety and depression [8]. Changes in these abilities in response to fluctuations in ovarian hormone levels may therefore inform our understanding of these disorders, and particularly their differing prevalence in men and women, as well as the onset of sex-specific disorders, such as Premenstrual Dysphoric Disorder (PMDD).

While there is considerable evidence from animal and human studies to suggest that ovarian hormones play a crucial role in social cognition [9,10], evidence for modulation of this role by menstrual cycle phase—especially in relation to aspects of mentalizing (i.e. facial affect recognition, empathy, etc.)—is currently sparse. Studies reporting an association between ovarian hormone levels and emotion recognition suggest that progesterone levels are negatively correlated with general accuracy when performance across all facial emotion expressions is considered [11,12]. Related studies also suggest that progesterone modulates subjective evaluations of emotion-intensity and attentional bias towards emotional signals [7,13]. The relationship between estrogen levels and emotion recognition, as well as the other emotional-evaluative indices found to be related to progesterone (i.e. emotion-intensity evaluations; attentional bias), remains unclear however, with only one previous study reporting an associated between estradiol and emotion recognition (anger recognition accuracy) [14].

Existing studies examining the effects of ovarian hormones on emotion recognition (see also [7,15,16]) have tended to only report a limited set of emotion recognition indices, particularly correct responses. This is a significant limitation because social decision-making often occurs under time-constraints and in the context of risk and uncertainty. Since social signals transmitted through facial expressions are inherently ambiguous, it is essential to model the uncertainty within which this decision-making occurs through the use of appropriate signal detection analytic methods. It remains possible that the resulting indices of performance, namely discrimination (sensitivity) and response bias may reveal associations with hormone levels and/or differences across menstrual phases not found in previous studies. Moreover, previous studies have used static stimuli whereas facial expressions are actually dynamic social signals transmitting information over time. Therefore in order to model ordinary social interactions more accurately, dynamic facial stimuli offer a more valid representation of social cognition in humans [17].

In the current study we extend previous research on the modulation of facial emotion recognition by ovarian hormones. Firstly, in addition to reporting number of correct responses, we assess reaction time and use a variant of signal detection analysis which controls for non-random guessing. As such we aimed to provide a more complete characterisation of the effects of hormonal levels and menstrual phase on emotion recognition. Secondly, while we primarily report the effects of ovarian hormone levels, we also provide a preliminary exploration of emotion recognition during three well-defined epochs in the menstrual cycle, including two separate intervals in the luteal phase (early and late). These intervals are of particular interest because they may have special relevance to susceptibility to PMDD in vulnerable individuals. Finally, we use photorealistic dynamic stimuli of facial expressions in our emotion recognition task [18,19].

## Materials and Methods

The research was approved by the University College London/University College London Hospital Research Ethics Committee.

### Procedure

Demographic details, weight and height were recorded before a saliva sample was taken. Questionnaires were then completed in a fixed order. The emotion recognition task (Dynamic Emotion Expression Recognition Task; DEER-T) was then performed, after which an unrelated involuntary memory task was completed as part of a separate study. All participants gave written informed consent and were compensated for their time.

### Participants

Forty-five healthy women aged 18–35 years were originally recruited via online advertising. Using an online screening assessment, participants were required to indicate that they were regularly cycling, with cycle length of 26–34 days and were not using any hormone-based contraceptives. Previous use of hormonal contraceptives must have stopped at least three months prior to participation. Participants were also required to declare an absence of psychiatric disorder. During the screening participants indicated their cycle duration and the start date of their last cycle (the first day of menses). They were contacted on the expected date of their new cycle to confirm if/when menses had started. Participants’ cycle phase at time of testing was based on this date and their cycle duration. For cycle lengths longer or shorter than 28 days, cycle phase classification was adjusted accordingly [20]. Testing was scheduled such that there were similar numbers of participants in each of the three periods of interest within the menstrual cycle: late follicular (days 7–13, *MED* = day 9.; n = 16), early luteal (days 15–19, *MED* = day 17; n = 14), and late luteal (days 22–27, *MED* = day 24; n = 14. This group was initially n = 15 but one participant’s data was excluded from all analysis; see statistical analysis section). The total sample size was in line with that required to detect a small-medium effect (f = 0.2) in a repeated measures analysis of within (expression)-between (cycle phase) factors interaction with an error probability (α) of 0.05 and power (1-β) of 0.80, assuming a correlation of 0.5 for the within subjects factor (required sample size: n = 36) [21].

Testing started at various times of the day, depending on participant availability between 10.00 and 18.00 hr. While there are diurnal variations in ovarian hormone levels [22], peak levels tend to occur early in the morning (between 6.00 and 9.00), with minimal variations in levels during the interval of the day when testing occurred for this study [22].

### Questionnaire measures

Dispositional anxiety, anxiety sensitivity, and premenstrual symptoms were assessed using the trait version of the State-Trait Anxiety Inventory (STAI-T) [23], the Anxiety Sensitivity Index (ASI)[24] and Premenstrual Tension Rating Scale-Updated Version (PMTS)[25] respectively. Positive and negative affect were assessed using the Positive and Negative Affect Schedule (PANAS) [26].

### Hormone analysis

Saliva samples were taken from passive drool (SaliCaps, IBL, Hamburg, Germany) and stored at -80°C until transportation on dry ice for analysis. Progesterone and estradiol levels were analyzed using luminescence immunoassay kits purchased from IBL International (Hamburg, Germany). Multiple control samples were run to receive the following coefficients of variation for progesterone: 7% for low (60 pg/ml), 11% for higher (260pg/ml) concentrations, and estradiol: 7% (3.7 pg/ml) and 9% (14.6 pg/ml). The sensitivities of the assays were 2.6 pg/ml and 0.3 pg/ml for progesterone and estradiol, respectively.

### Emotion recognition task

The task used here—the DEER-T—is sensitive to neuropharmacological effects ([19], [18]) and comprises dynamic video-like stimuli which are intended to simulate the non-instantaneous occurrence of emotion expressions in social situations. Static colour photographs of twelve Caucasian actors (six women) were morphed using Abrosoft Fantamorph software (version 4.0) to create dynamic changes in facial expressions from neutral to five target emotions: anger, happiness, and fear with mouth open; sadness and disgust with mouth closed. Dynamic neutral expressions were also created by morphing from neutral mouth open to neutral mouth closed. Stimuli were taken from the NimStim set of facial affect [27]. The resulting ‘videos’ had a duration of 3000 ms. Six labelled response keys corresponded to the six expression. To reduce working memory load, a diagram of the response keys in relation to finger locations was displayed on the computer monitor directly below the presented stimuli. Participants were instructed to press the key corresponding to the correct emotion and to respond as quickly and accurately as possible. Our recent study suggests that the task has excellent psychometric properties[18].

The DEER-T began with 12 practice trials consisting of neutral → sad, happy, angry, fearful, disgusted and neutral expressions each displayed twice. Trials began with a black fixation cross for 1000 ms in the centre of a white screen, followed by a stimulus. A key press ended the trial and immediately began the next. All participants were sat approximately 40 cm from a 17-inch (43.2 cm) desktop computer monitor through which instructions and stimuli were presented. Feedback was provided during practice trials to indicate whether responses were correct or not. If no response was made within 3000 ms, the words “TOO SLOW” appeared for 2000 ms followed by commencement of the next trial. Practice trials were not included in the analysis. For the main task, stimuli were presented in a similar manner. This consisted of two blocks of 72 trials (144 trials in total; 24 presentations of each expression) presented in pseudorandom order such that the same emotion was not presented more than twice in succession. No feedback was provided in the experimental trial.

### Statistical analyses

Data were initially inspected for outliers, defined as Z score-transformed values of >2.5 SDs. Data from one participant (in the late luteal group) with progesterone and estradiol levels >5 and 6 SDs respectively was thus removed from all analyses. Other data points identified as outliers according to the above definition were excluded listwise and eventual sample sizes for analyses after removal of outliers are reported separately for each performance index (correct responses, reaction time, discrimination and response bias) in the repeated measures analyses. Data was inspected for normality and transformations applied where distributions were non-normal to enable the use of parametric statistics. In particular, progesterone levels were positively skewed so were log transformed, as in previous studies [7,28]. Untransformed progesterone levels are reported in Table 1 for ease of comparison with previous studies but transformed values were used in all analyses.

**Table 1:** Demographic, endocrine and mood-related characteristics of the follicular (n = 16), early luteal (n = 14) and late luteal (n = 14) cycle phase groups (mean ± SD).

	Follicular (days 7–13)	Early luteal (days 15–19)	Late luteal (days 22–27)
**Age**	22.50 ± 3.37	23.54 ± 4.26	23.13 ± 3.58
**Years Education**	16.00 ± 1.92	15.69 ± 2.43	15.73 ± 2.12
**Progesterone (pg/ml)**	72.61 ± 47.72	125.95 ± 124.24	211.86 ± 181.36
**Estradiol (pg/ml)**	5.71 ± 1.98	6.11 ± 2.57	5.79 ± 2.34
**Anxiety Sensitivity**	27.56 ± 9.06	29.07 ± 9.08	27.21 ± 12.82
**Premenstrual symptoms**	52.13 ± 21.65	50.71 ± 18.49	50.36 ± 18.84
**Trait anxiety**	47.63 ± 3.86	44.50 ± 6.20	47.36 ± 3.86
**PANAS positive**	32.38 ± 6.81	31.64 ± 5.81	34.14 ± 6.32
**PANAS negative**	23.00 ± 7.33	21.00 ± 7.97	23.07 ± 7.30

We applied a variant of signal detection analysis to determine discrimination (P_r_) and response bias (B_r_) as described in [2]. P_r_ and B_r_ are variants of the d’ and C indices used in signal detection analysis. The two-high threshold approach is appropriate when there are unequal numbers of targets and distracters, as in the DEER-T (see [29]). Discrimination (P_r_) refers to the ability to discriminate one emotion from others. It is calculated using the equation:

$$P_{r}=HR\;-\;FAR$$

where HR and FAR are the adjusted Hit and False Alarm Rates respectively. Response bias (B_r_) is a systematic tendency to respond to specific targets in a particular way and can be classified as liberal or conservative. It is derived as follows:

$$B_{r}=\frac{FAR}{1-\left(HR-FAR\right)}$$

In the context of the current study, a liberal response bias might involve a systematic tendency to respond ‘angry’ when other facial expressions are presented. As such even a mildly furrowed brow or any other sign of distress is perceived and responded to as angry. Higher values of B_r_ suggest a liberal response bias. Conservative responding on the other hand involves treating equivocal stimuli as ‘safe.’ In the real world this tendency might arise when incorrect identification of a stimulus as threatening would entail risks or costs (for example, causing embarrassment if sadness was misperceived as anger) (see [30]). This approach is described in further detail in Pollak et al [2].

Data were analysed using the Statistical Package for Social Sciences (Version 21; IBM, California, USA). Correlation coefficients (Pearson’s *r*) expressing the relationship between hormone levels and emotion recognition performance were determined for overall emotion recognition and separately for each facial expression. Correlation analysis was not further separated by stimulus gender or menstrual phase to avoid an unacceptable high false positive rate. No adjustment was applied to the alpha level in the correlation analyses (although if the adjusted alpha level of α<0.008 was used, most of the flagged correlations in Table 2 would remain significant). One-way Analysis of Variance (ANOVA) was used to compare cycle phase groups on baseline demographic, mood and hormonal variables. A series of 3 x 6 repeated measures ANOVAs were used to examine the effect of cycle phase (between subjects factor) on the facial expression recognition (within subjects factor) across four dependent variables (number correct, RT, discrimination and response bias). When sphericity could not be assumed Huynh-Feldt corrected values were used.

## Results

### Participant characteristics

Key participant characteristics are summarised in Table 1. There was no difference in these parameters, or in measures of anxiety sensitivity, trait anxiety, premenstrual symptoms, positive and negative affect between the follicular, early luteal and late luteal phase groups (F values <1.0, p values>0.4).

In line with purposive sampling at the specific periods of the menstrual cycle of interest, progesterone levels differed significantly between cycle phases (F_2,43_ = 5.226, p = 0.01). Post-hoc analysis showed that follicular phase progesterone levels were significantly lower than the late luteal phase (p = 0.002).There was no difference across cycle phases in estradiol levels (F_2,43_ = 0.123, p>0.5). Progesterone and estradiol levels were not correlated with each other or with the pre-menstrual symptoms, anxiety, positive or negative affect (p values >0.2).

### Facial expression recognition performance and salivary ovarian hormone levels

Associations between estradiol and progesterone and the facial expression recognition indices are displayed in Table 2.

**Table 2:** Pearson’s correlation coefficients expressing the relationship between progesterone^1^ and estradiol and indices of emotion recognition performance for six facial expressions.

		Reaction time	Number correct	Response bias	Discrimination
**Progesterone**	**Anger**	0.38*	-0.16	0.02	0.01
	**Disgust**	0.25	0.05	0.27	0.05
	**Fear**	0.29	-0.26	0.02	-0.24
	**Happiness**	0.40**	-0.03	-0.06	0.09
	**Sadness**	0.51***	0.00	-0.09	0.11
	**Neutral**	0.42**	-0.20	-0.32*	-0.10
**Estradiol**	**Anger**	0.10	-0.13	0.17	-0.26
	**Disgust**	0.27	-0.45**	-0.34*	-0.43**
	**Fear**	0.25	0.05	-0.02	0.05
	**Happiness**	0.16	0.05	0.21	-0.11
	**Sadness**	0.19	0.07	0.06	0.16
	**Neutral**	0.28	-0.22	-0.21	-0.18

^1^Log transformed values were used although the pattern of results was the same with untransformed progesterone levels.

Sample = 42–44 throughout

*<0.05.

**<0.005.

***<0.001.

**Fig 1:**
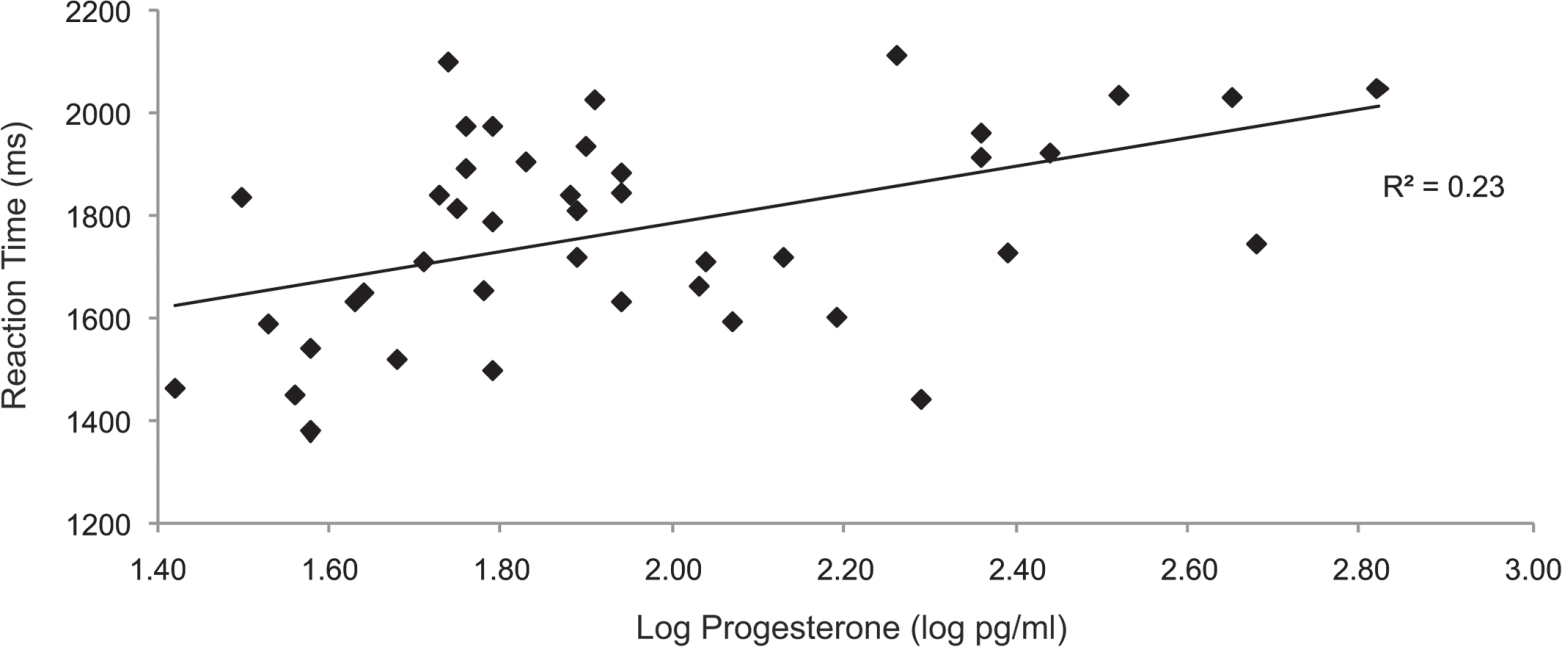
The association between reaction times across all emotions and progesterone (log) values. Line of best fit is shown along with R^2^ indicating the degree of variance in recognition performance accounted for by progesterone.

Previous studies have reported associations between progesterone levels and performance averaged across all emotions [11,12] rather than specific expressions. Here we also found that progesterone levels were positively correlated with reaction times averaged across all facial expressions (*r*(44) = 0.475, p = 0.001; Fig 1). Progesterone levels did not correlate with response bias (B_r_), discrimination (P_r_), or correct responses collapsed across all facial expressions. We also assessed associations between progesterone and individual facial expressions as shown in Table 2. There were significant positive correlations between progesterone and reaction times to sad, neutral, happy, and angry expressions and a trend-level correlation with fearful (p = 0.054), but not disgusted expressions (p = 0.114; Table 2). Higher progesterone levels therefore predicted a generalised slowing of reaction times. A negative correlation between progesterone levels and response bias to neutral expressions was also found (Table 2). Progesterone levels were not associated with arousal items ‘difficulty concentrating’ and ‘lethargy’ on the PMTS (p values > 0.3) suggesting that progesterone effects on reaction times were not mediated by arousal. In contrast to progesterone effects, as shown in Table 2, estradiol levels did not correlate significantly with reaction times for any expression (p≥0.062).

**Fig 2:**
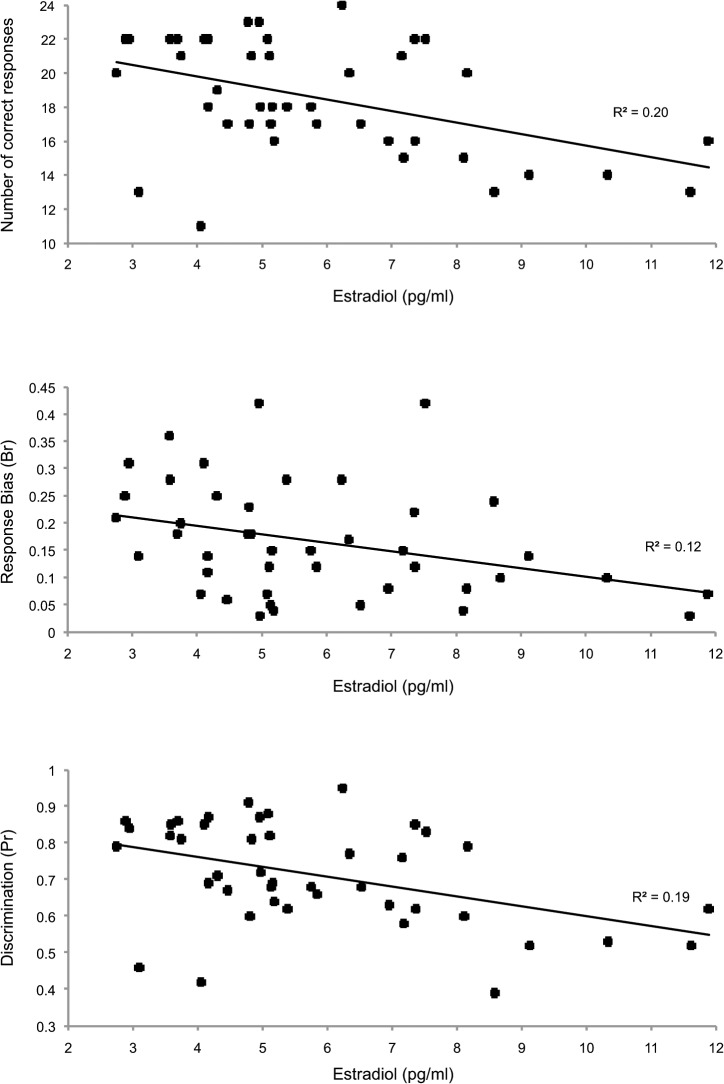
. Association between estradiol (pg/ml) and disgust recognition indices. Lines of best fit are shown along with R^2^ indicating the degree of variance in recognition performance accounted for by estradiol.

Estradiol levels correlated (negatively) with correct responses across all expressions (r = -0.349, p = 0.02), but not overall B_r_ (r = -0.082, p = 0.619), P_r_ (-0.181, p = 0.276), or reaction times (r = 0.265, p = 0.082). However, significant negative correlations were found between estradiol levels and correct responses, response bias (B_r_) and discrimination (P_r_) of disgust expressions (Fig 2). In contrast, as can be seen in Table 2, disgust recognition performance indices were not significantly associated with progesterone levels (r≤0.27; p≥0.086).

### Emotion recognition performance across cycle phases

Given our previous observation of pronounced differences in emotional processing (distressing, intrusive emotional memories) between early and late luteal phases [31], we examined whether there were interactions between cycle phase and expression in a series of repeated measures ANOVAs.

Analysis of correct recognition of emotion expressions showed a main effect of expression (F_3.90, 136.42_ = 39.556, p<0.0001, η_p_^2^ = 0.531; n = 38), with happiness recognition at ceiling level (mean % correct = 95.83±1.48), and sadness having the lowest level of correct identification across groups (71.21 ± 13.80% correct). There was no main effect of cycle phase (F_2,35_ = 0.172, p = 0.842) and no cycle phase x expression interaction (F_7.80, 136.42_ = 0.938, p = 0.486, η_p_^2^ = 0.051).

Reaction times showed a large effect of expression (F_5,195_ = 80.426, p<0.0001, η_p_^2^ = 0.673; n = 42) with happy faces recognised most rapidly (1359.75 ± 196.72 ms) and all other expressions recognised within the ~1800–1950 ms range. There was no effect of cycle phase (F_2,39_ = 0.001, p = 0.999, η_p_^2^ = 0.00) and no cycle phase x expression interaction (F_10,195_ = 0.566, p<0.84, η_p_^2^ = 0.028).

Analysis of response bias (B_r_) showed a main effect of expression (F_3.96, 142.53_ = 11.259,p<0.0001, η_p_^2^ = 0.249; n = 39), no effect of cycle phase (F_2,36_ = 0.389,p = 0.681, η_p_^2^ = 0.021) and no interaction between these factors (F_7.92,142.53_ = 1.183,p = 0.314, η_p_^2^ = 0.062). Similarly, discrimination (P_r_) showed a main effect of expression (F_4.12, 144.01_ = 46.183, p<0.0001, η_p_^2^ = 0.569; n = 38) with lower P_r_ values for sadness. However, there was no effect of cycle phase (F_2,35_ = 0.266, p = 0.768, η_p_^2^ = 0.015) and no cycle phase x expression interaction(F_8.23, 144.01_ = 0.865, p = 0.550, η_p_^2^ = 0.047).

When these analyses were repeated to only include follicular and *either* early *or* late luteal phases or follicular and early and late luteal phases combined, there was similarly no evidence of main/interaction effects involving cycle phase (all F values <1.7, all p values >0.1).

## Discussion

This study aimed to provide a comprehensive description of the effects of ovarian hormones on emotion recognition in naturally cycling women tested during one of three epochs of the menstrual cycle. Primarily we found a generalised association between progesterone and reaction times to facial expressions and a more specific association between estradiol and disgust processing. Specifically, higher progesterone levels were associated with slowing of responses to expressions of anger, sadness, happiness and neutral expressions. On the other hand, the negative association between estradiol levels and correct responding, discrimination and response bias to disgust expressions indicated that higher estradiol levels—which occur just prior to ovulation—were associated with *lower* correct recognition, discrimination and response bias to disgust. Exploratory between groups (cycle phases) analyses showed no evidence of differences across the three studied cycle phases.

Correlation coefficients expressing the relationship between progesterone and reaction times for neutral, sad, happy and angry expressions were statistically significant and indicated medium to large effects. It is possible that the lack of significant associations with fear and disgust may reflect insufficient power rather than specificity of an association with the other expressions. Although we did not have a non-emotion-related control task, the association between progesterone and reaction times to neutral (non-emotional) expressions might suggest that slowing of reaction times at high progesterone levels is not specific to emotional processing *per se*. Some research supports the idea that progesterone affects processing of human faces as biologically salient stimuli. For example, oral progesterone administration in healthy women reduced activity in the fusiform gyrus [32] and was also associated with functional uncoupling of fusiform gyrus and amygdala during the encoding phase of a facial expression-memory task [33]. Further support for a generalised (non-emotion-specific) effect on face processing by progesterone is the finding that progesterone levels are correlated (negatively) with amygdala activity to sad and fearful emotional expressions but also to neutral faces [34]. Such reduced activity may underlie the slower reaction times at higher progesterone levels seen here or in reduced general emotion recognition accuracy observed in previous studies [11,12].

One previous study has shown that progesterone levels are associated with disgust (and fear) processing but only when stimuli were of facial expressions with averted eye gaze [13]. This specific effect (no association was found with facial stimuli with direct eye gaze) suggests that high progesterone may be linked to communicating the threat of contamination to others [35]. We are not aware, however, of any study reporting an association between estrogens and disgust processing as reported here. Our findings are intriguing in light of studies showing that estrogenic processes are involved in pathogen avoidance through non-verbal communication of contagion-threat in rodents [9]. Adaptationist accounts of the role of disgust in humans extend its role beyond disease-avoidance and dietary-selectivity to, for example, sexual behaviours/selectivity, which varies across the menstrual cycle [36]. In considering the relevance of our findings to such accounts it is worth noting that we used direct-gaze facial expression stimuli. Direct-gaze expressions of disgust have a specific communicative function, expressing moral violation/contempt. This is distinct from the function of averted gaze expressions of disgust, which, as implied above, express contamination threat (e.g. [13]). In future studies, a comparison between responses to averted- and direct-gaze expressions of disgust would be informative since our findings, and those of Conway and colleagues [13], suggest that perception of disgusted faces may be differentially modulated by progesterone and estrogens respectively depending on direction of gaze.

Participants in the current study were purposively sampled at specific intervals in the menstrual cycle: mid follicular, early luteal and late luteal phases. The latter is characterised by pronounced endocrine, physiological and subjective-mood changes in the majority (up to 75%) of healthy women sampled from the general population [37] which may be reflected in changes in social cognition. In addition however, the early luteal period may also be relevant to symptoms of psychopathology, since women in this phase appear to show an enhancement of distressing involuntary emotional memory, a transdiagnostic symptom in psychological disorders [31]. While we did not find evidence of cycle phase effects in the current study, a number of factors may explain these null findings. In particular, while we ostensibly obtained an adequate sample size to detect medium effects with power (1-β) = 0.8, it is possible that effects were too small to be uncovered using a significance testing approach. Although previous studies have found between-phase effects with similar (or smaller sample sizes) we cannot rule out the possibility that responses to dynamic expressions are different (with a smaller effect size) than those found with static images used in previous studies.

Additional limitations in our study must also be acknowledged. Firstly, a repeated measures, within subject design, with each participant attending on separate occasions across the three phases may have reduced error variance. On the other hand, such a design would need to consider the impact of practice effects. Secondly, while progesterone and estradiol levels reported here were in line with those in previous studies that have sampled the same periods in the menstrual cycle [38], and we carefully tracked the menstrual cycle using a commonly-used diary method that involved self-tracking, additional confidence in the grouping of individuals according to the three phases could have been achieved using an assessment of ovulation. This is suggested as a further refinement to future studies.

## Supporting Information

S1 Datafile (SAV)
